# Level and factors associated with physical activity among university teacher: an exploratory analysis

**DOI:** 10.1186/s13102-021-00346-5

**Published:** 2021-09-25

**Authors:** Katarzyna Kwiecień-Jaguś, Wioletta Mędrzycka-Dąbrowska, Monika Kopeć, Renata Piotrkowska, Katarzyna Czyż-Szypenbejl, Rita Hansdorfer-Korzon, Magdalena Lemska, Piotr Jarzynkowski

**Affiliations:** 1 Department of Anaesthesiology and Intensive Care Nursing, Nursing and Midwife Institute, Medical University of Gdańsk, Gdańsk, Poland; 2 Department of Human Nutrition, University of Warmia and Mazury, Olsztyn, Poland; 3 Department of Surgical Nursing, Medical University of Gdańsk, Gdańsk, Poland; 4 Department of Physical Therapy, Medical University of Gdańsk, Gdańsk, Poland; 5 Department of Social Medicine, Medical University of Gdańsk, Gdańsk, Poland

**Keywords:** Physical activity (PA), IPQA, GSE, University teachers, Medical University, Technical University

## Abstract

**Background:**

Physical inactivity is one of the primary factors that leads to obesity and overweight. What is more, it is becoming an increasingly common problem among the population of those who work. The causes of obesity and the lack of physical activity are multifactorial. The aims of the study were: to (1) measure the level of physical activity among the university staff, (2) evaluate what factors have a significant influence on undertaking the physical activity and lack of.

**Methods:**

A cross-sectional study was conducted via the Internet questionnaires among university staff in Northern Poland and Pomeranian Region. Taking into consideration the climate and cultural factors in Poland, a physical activity test with the usage of IPAQ scale was performed between September and November 2018 and between March and June 2019. The data was collected on the basis of the standardized long form of the IPQA questionnaire, the GSE Scale and the interview questionnaire including questions about sex, age and health assessment.

**Results:**

The study group consisted of 276 respondents, including 143 women (51.8%) and 133 men (48.2%). The average age of the respondents was 42.22 with SD ± 11.01. The weight status was categorized with the use of BMI index. 51.3% (142) of the respondents had normal body mass, 93 (33.8%) were overweight, while 23 (8.4%) were obese. The mean BMI index was 25.23 points (SD ± 4.04). One hundred and twenty five (45.1%) respondents were the employees of the Medical University, and 54.9% were employed by the Technical University. Detailed analyses using Spearman correlation test confirmed the presence of a statistically higher level of physical activity among respondents employed at the Medical University (M 513.37; SD ± 609.13) than the employees of Technical University (M 378.38; SD ± 328.26). The odds ratio analysis shows that a low level of physical activity in the group of technical university staff has a significant correlation with the other social behavior which is the alcohol consumption.

**Conclusion:**

This study confirms that the number of points obtained in the IPAQ scale, classified the academics in the workgroup presenting low physical activity which does not exceed 600 MET-min/week. The most common form of activity in this group was walking. There was no correlation between physical activity and self-efficacy, age, marital status or the number of children.

## Background

Low level of physical activity remains currently the greatest public health problem of a modern society. It has been estimated that over half of the adult citizens of Europe, United States of America and Australia lead a sedentary lifestyle and undertake physical activity on the irregular basis only [1, 2] Yet, it is the physical activity that is a key factor to maintain physical, mental health and normal cognitive processes [3]. Environmental changes associated with the lack of physical activity often result in becoming overweight which is accompanied by diseases [4], including arterial hypertension, diabetes, stroke, ischemic heart disease and colon cancer [5, 6]. Regular physical activity increases exercise capacity and can lead to many health benefits such as quality of life [7] and general well-being. Physical activity undertaken by middle-aged adults or elderly people is significantly, positively correlated with cardiopulmonary efficiency, strength, flexibility, reduction of anxiety and depression symptoms, improvement of the sleep quality [8]. The participation in regular physical activity gives numerous benefits while those changes depend on the intensity of effort, duration and frequency [9].

The recommendations on the quantity and quality of physical effort vary for particular age groups and may differ between populations as well. The WHO guidance says that healthy adults aged 18–64 years should get at least 150–300 min of a moderate-intensity physical activity or 75–150 min of vigorous-intensity physical activity per week. To increase additional health benefits, one should develop a moderate intensity PA to more than 300 min per day [8]. Another method which would allow getting adequate dose of physical activity is walking 10,000 steps per day [10]. When it comes to adults, general recommendations state that the optimal energy expenditure is 1500–3500 kcal/week. People should be encouraged to undertake physical activity. However, when studying the adult population, it is still difficult to provide precise estimations when it comes to the energy expenditure and its correlation with the forms of physical activities that are undertaken. There is a very strong need to precisely determine the energy expenditures in specific areas of physical activity, including spare time, professional work, working at home and transportation [11, 12]. Another very important aspect related to undertaking physical activities is a healthy lifestyle which may be described as a set of everyday decisions, habits and behaviours. Apart from physical activity it is worth mentioning other crucial elements such as: proper diet, avoiding alcohol and nicotine, ability to cope with stress [13]. The participation in the healthy lifestyle programs is associated with many social and environmental aspects. They have been divided by the researchers into the independent factors like: sex, age, race, ethnicity as well as behavioral factors including personality, environmental and social aspects [14].

Many studies examine the physical activity among cardiac and oncological patients’ populations, elderly, adolescents, children population, students or people with low socioeconomic status. Only a few studies have investigated the problem among the university staff although this population seems to be more familiar with the benefits of physical activity, especially teachers who are the specialist in their fields [15, 16]. The knowledge, however, is not always followed by the action. Lack of time, low motivation and fatigue are very often the biggest obstacles. The research conducted at the University of Minnesota confirmed that more than a half of employees does not take any special physical activities. The authors stated that workers who demonstrate “sedimentary work activities” eventually reduce their non-work physical activity. The study confirmed that the vast majority of university staff spends more than 75% of their working time in a sitting position [15]. Kwan et al. suggest that the most significant decline in the PA among the population of the students entering the universities [17].

### Social cognitive theory and self-efficacy

About a decade ago, The Stanford University teacher Albert Bandura translated his basic research called social cognitive theory (SCT). This new theory provided more knowledge into the field of psychology. From that time people were viewed as a self-organizing, proactive, self-regulating and self-reflecting rather than reactive organisms. This new psychological theory led to the conclusion that human functioning seems to be a product of a dynamic correlation of personal, behavioral and environmental factors [18]. One of the elements of this theory is perceived self-efficacy, i.e. self-belief in own capabilities to efficiently accomplish activities, impact personal choices while facing barriers and failures [19]. This means that if somebody is sure that could walk a mile without any difficulty, the self-efficacy would be high and could have a huge impact on his/her behavior [20]. Badura’s theory suggests four main sources that have undoubtedly an impact on self-efficacy. These are past performance, personal experience, social precautions and psychological factors like the level of stress or anxiety [20]. The structure of Badura’s model can be helpful in applying the solutions to replace unhealthy habits with new behaviors in addition to explain what factors have a significant influence on that particular behaviors. It was proven that perceived self-efficacy influences the healthy attitudes and has a significant association with undertaking physical activity [21]. It is even recommended that before setting personal training goals it is worth to evaluate the level of self-efficacy. The low results of the evaluation process should be discussed with a potential client because it can decrease his/her attitude towards active living or any kind of physical activity [20].

Some research suggest that young adults who exercise regularly, show higher levels of perceived self-efficacy in overcoming barriers such as lack of time, distance to sport facilities, costs as well as shame which could hinder undertaking physical activity. Leganger and co-authors indicate that there is a strong correlation between self-efficacy measured with GSE scale and healthy behaviors [22]. Szczuka in her research mentioned that although more longitudinal studies are required, there is an association between self-esteem and sedentary behaviors [23].

The present study was the first research of that kind in Poland.

## Methods

The research was conducted with the use of a descriptive, comparative design. This method was used to describe and examine the differences between two groups that may occur naturally in the setting. The aims of the study were: to (1) measure the level of physical activity among the university staff, (2) evaluate what factors have a significant influence on the low level of physical activity (including self-efficacy, age, sex, professional position, marital status, number of children, and healthy behaviors).

### Sample and study design

Taking into consideration the climate and cultural factors in Poland, physical activity tested with the IPAQ scale has seasonal character. The study was performed in two periods of time, i.e. between September and November 2018 and between March and June 2019. 276 employees of Pomeranian universities, in the northern part of Poland, were enrolled into the project. The study included interview and questionnaire method using standardized International Physical Activity Questionnaire (IPAQ), and Generalized Self-Efficacy Scale (GSE). Socio-demographic information was collected with interview including such data as: sex, age, height, marital status, general health assessment, number of cigarettes smoked every day and use of other psychoactive substances.

### The procedure of the study

We have decided to invite three of the largest universities in northern Poland to recruit the teachers. Finally, only two were included in the study procedure although all university authorities agreed to participate in the project. The Medical University of Gdańsk (MUG) employs 1099 university teachers, whereas Technical University hires 1300 teachers in various academic positions. The response rate was from 11.37 to 11.69%. The use of the study tools was possible thanks to the consent issued by the owners of the respective copyrights. The invitation letter and the questionnaire were distributed via the Internet using the Google questionnaires platform. The respondents received an e-mail with the description of the project, its main assumptions, and a link to the platform with the questionnaire. The research team cooperated with the IT specialist from each university. The invitation letter with the link was distributed via the employees’ web domain. The questionnaire was distributed among the university teachers conducting didactic classes or science courses. When the participants agreed to take part in the project, they had to submit the information “I would like to take participate in the study and fill in the questionnaire”. In case of questions, the respondends could contact the research member via the e-mail address or the telephone.

### Study tools

#### Generalized Self-Efficacy Scale (GSE)

Generalized Self-Efficacy Scale refers to the concept of expectations and the idea of perceived self-efficacy which was developed by Shwarzer and Jerusalem [24]. The Polish version of the scale included 10 statements relating to the measurement of the efficacy manifesting itself in the specific situations that are problematic for the respondent. The respondent marks the answers by himself/herself. Each question has four answers to choose from, ranging from: “not at all true”, “hardly true”, “moderately true”, “exactly true” Each answer is given the appropriate number of points (1 to 4). The total score, being a sum of all the points, reflects the general indicator of self-efficacy. The higher the total score, the greater the level of perceived self-efficacy [25]. The scale was chosen because some research suggests that self-efficiency is strongly correlated with healthy attitudes and may have impact on physical activities [22–25].

#### International Physical Activity Questionnaire (IPAQ)

The International Physical Activity Questionnaire (IPAQ) measures a habitual practice of physical activities of populations in different countries based on the socio-cultural contexts. The scale become the most widely used physical activity survey. The two forms of IPAQ have been developed: a short and a long version. The long version, used in the project, was designed to provide a comprehensive evaluation of daily physical activities. Moreover, it was chosen to assess the time spent walking, doing moderate or vigorous intensity activity. The scale analyses three aspects of physical activity, namely the frequency, the duration and intensity in the last 7 days. The physical activity level is expressed in MET-min/week (MET—*Metabolic Equivalent of Task*). It allowed to unambiguously allocate the respondents to one of the three categories of activity: Low—no activity reported, or some activity is reported but it is insufficient to meet the moderate or high categories. Moderate physical activity—3 or more days of vigorous activity for at least 20 min per day, or 5 days or more of moderate intensity activity, or walking at least 30 min. per day or 5 or more days of any combination of walking, moderate or vigorous intensity activities achieving a minimum 600 MET-min/week. High—3 or more days of vigorous activities accumulating at least 1500 MET-min/week, or 7 or more combinations of walking, moderate or vigorous activities achieving a minimum of 3000 MET min/week [26, 27].

MET unit is a metabolic equivalent corresponding with the oxygen consumption at basal metabolic rate. The analysis of the scientific study allowed specifying that 1 MET is equivalent to 3.5 ml O_2_/kg body mass/min. Vigorous intensity physical activity was defined as greater than 6 MET for each minute of its duration. Moderate intensity physical activity equals 3–6 METs [26, 27].

#### BMI index

The Body Mass Index (BMI) was calculated on the basis of a self-reported body weight and body height. Next, the BMI was calculated using the simple formula—weight (kg) divided by the square of the body height (m^2^). BMI was also categorized using international WHO adult standards: underweight—BMI < 18.5, normal weight—BMI = 18.5 to 24.9, pre-obesity—BMI = 25.0 to 29.9, and obesity class I—BMI 30.0–34.9, obesity class II—BMI 35.0–39.9, obesity class III—BMI above 40.0 [28].

### Ethical committee approval

The project was approved by the Bioethical Committee for the Scientific Research of the Medical University of Gdańsk (No. NKBBN/36/2018), as well as by the chancellors of the universities which participated in the study. The subjects participating in the study were informed about the aim and the assumptions of the study. Respondents were guaranteed full anonymity. Questionnaire was distributed via the Internet, participating subjects did not have to log on or give any personal data in relation to the project. The respondents gave their written on-line consent to participate in the project. The researchers prepared an on-line version of a consent form. The consent was given by clicking a button “Yes, I would like to fill in the on-line questionnaire”. If the respondents decided to take part in the research they were transferred into the questionnaire platform. The answers of the respondents were secured using a virtual “Cloud” database.

### Statistical analysis

All statistical calculations were carried out using the IBM SPSS 23 statistical package and an Excel 2016 spreadsheet.

Qualitative variables were presented as numbers and percentages, while quantitative variables were characterized using arithmetic mean and standard deviations. The significance of any differences between more than two groups was verified using the Kruskal–Wallis non-parametric significance test. When the data was not obtained confirmation of normal distribution. The significance of differences between the two groups was tested by using the Mann Whitney U test and Student t test. Spearman correlation test was used to verify the existence, the power and the direction of the relationship between the variables. For the purpose of establishing which factors were potentially related to the low physical activity, the odds ratios (OR) and its 95% confidence intervals (95% CI) were analysed. In all calculations, *p* < 0.05 was assumed as the level of significance.

## Results

The study group consisted of 276 respondends, including 143 women (51.8%) and 133 men (48.2%). There were one hundred and forty subjects (50.7%) with Ph.D. degree, 65 associate professors (dr hab.) and 22 professors (8.0%). As much as 260 (94.2%) subjects were academics. One hundred and twenty five (45.1%) respondends were employees of the Medical University, and 152 (54.9%) were employed by the Technical University. The Medical University of Gdańsk (MUG) is the largest Medical University in the Northern Poland. The MUG educates more than 6000 undergraduates and postgraduate students at 4 Faculties: Faculty of Health Sciences, Faculty of Medicine, Faculty of Pharmacy and the Intercollegiate Faculty of Biotechnology. The Technical University is one of the 4 places among Polish Universities. It educates students from more than 70 countries at 9 Faculties. The mean age of the respondends who participated in the project from both universities was 42.22 years (SD ± 11.01).

One of the last questions referred to an assessment of a general health status: 36.6% of the subjects assessed their health as very good, 41.7% (115) as good, while 16.7% (46) as rather good. Basing on the given data BMI was calculated for each subject, and then they were allocated to categories created according to basic WHO classification. The analysis of the collected data revealed that more than 51.6% (142) of the respondends had normal body mass, 93 (33.8%) were pre-obesity, while 11.7% were obese. More detailed characteristic of the studied group is presented in the Table 1.

**Table 1:** Socio-demographic characteristic of the studied group

Variables	Categorial						
	Medical University		Technical University		All		*p* value
	n	%	n	%	n	%	
*Sex*							
Female	89	32.2	54	19.6	143	51.8	0.010*
Male	36	13.0	97	35.2	133	48.2	
*Scientific title*							
Professor	8	2.9	14	5.1	22	8.0	0.097
Associate professor (dr hab.)	22	8.0	43	15.6	65	23.6	
Ph.D	72	26.1	68	24.6	140	50.7	
M.Sc	23	8.3	26	9.5	49	17.8	
*Post*							
Research fellow	4	1.4	12	4.4	16	5.8	0.093
Academic	121	43.8	139	50.4	260	94.2	
*Smoking*							
10–20 per day	3	1.1	5	1.8	8	2.9	0.596
< 10 per day	6	2.2	3	1.1	9	3.3	
Ex-smoker	11	4.0	14	5.1	25	9.1	
Non-smokers	105	38.0	129	46.8	234	84.8	
*Alcohol consumption*							
Once a day	2	0.7	4	1.5	6	2.2	0.099
Few times in a week	16	5.8	22	8.0	38	13.8	
Few times in a month	86	31.1	83	30.1	169	61.2	
Never	21	7.6	42	15.2	63	22.8	
*General assessment of health*							
Poor	1	0.4	4	1.4	5	1.8	0.140
Rather good	17	6.2	29	10.5	46	16.7	
Good	48	17.4	67	24.3	115	41.7	
Very good	53	19.2	48	17.4	101	36.6	
Excellent	6	2.2	3	1.1	9	3.3	
*Marital status*							
Single	23	8.3	34	12.4	57	20.7	0.256
Married	98	35.5	105	38.1	203	73.6	
Divorced	3	1.1	10	3.6	13	4.7	
Widowed	1	0.4	2	0.7	3	1.1	
*Number of children*							
0	42	15.2	57	20.7	99	35.9	0.199
1	20	7.3	28	10.1	48	17.4	
2	54	19.6	47	17.0	101	36.6	
3	8	3,0	14	5.0	22	8.0	
4	1	0.4	5	1.8	6	2.2	
*General assessment of physical activity*							
Low	100	36.2	122	44.2	222	80.4	0.038
Moderate	15	5.4	26	9.4	41	14.8	
High	10	3.6	3	1.1	13	4.7	

^*^The level of significance Chi^2^*p* = 0.05

### GSE scale

The analysis of the reliability of questionnaire scales calculated with Cronbach’s alpha coefficient (α = 0.870) determined the consistency of the positions of the GSE scale as satisfactory. The general index of self-efficacy obtained by summing up all the points from the scale allowed calculating the mean score for the studied population, which was 31.52 (SD ± 3.71).

The sum of all the points obtained in GSE scale allows calculating general self-efficacy score, which ranges from 10 to 40 points. The mean score of 31.52 (SD ± 3.71) indicates the high level of perceived self-efficacy of the respondends (Table 2).

**Table 2:** GSE scale

GSE no question	MIN	MAX	M	SD
1. I can always manage to solve difficult problems if I try hard enough	1	4	3.28	0.52
2. If someone opposes me, I can find the means and ways to get what I want	1	4	2.91	0.54
3. It is easy for me to stick to my goals and accomplish them	1	4	2.98	0.57
4. I am confident that I could deal efficiently with unexpected events	1	4	3.07	0.50
5. Thanks to my resourcefulness I know how to handle unforeseen situations	1	4	3.17	0.56
6. I can solve most problems if I invest the necessary effort	1	4	3.33	0.53
7. I can remain calm when facing difficulties because I can rely on my coping abilities	1	4	3.08	0.66
8. When I am confronted with a problem, I can usually find several solutions	1	4	3.11	0.56
9. If I am in trouble, I can usually think of a solution	1	4	3.26	0.49
10. I can usually handle whatever comes my way	1	4	3.33	0.52

Min—minimal, Max—maximal, M—mean, SD—standard deviation

### Physical activity—IPAQ scale

The first part of the questionnaire was job-related (paid work, voluntary work, while doing course work as well as unpaid work away from home).

The analysis of respondends’ answers reveal that job-related vigorous-intensity activities were performed for a mean of 1 day (M 0.80; SD ± 1.49). During one of these days vigorous-intensity activity lasted 14.04 min on average (SD ± 34.62). Job-related moderate-intensity activity was performed for a period of over one day (M 1.23; SD ± 1.84) (Table 3). A total number of minutes spent on moderate-intensity activity was 27.70 min (SD ± 54.67). The studies show that walking took the most of the time during one day, mean 52.79 min (SD ± 2.47). Detailed analyses are presented in Table 3.

**Table 3:** Descriptive statistics—physical activity: work-related, transportation-related, domestic, recreation and sport, time spent sitting

	N	MIN	MAX	M	SD
*Physical activity—work-related activities*					
Vigorous-intensity physical activities (days)	151	0	7	0.80	1.49
Vigorous physical activities (min)	148	0	240	14.04	34.62
Moderate-intensity physical activities (days)	217	0	7	1.23	1.84
Work-related moderate-intensity physical activities (min)	213	0	360	27.70	54.67
Walking for at least 10 min (days)	231	0	7	3.03	2.47
Total time walking on those days (min)	233	0	1200	52.79	97.92
*Physical activity—transportation-related activities*					
Travel in a motor vehicle (days)	268	0	7	5.60	1.91
Time spent travelling in a motor vehicle on one of those days (min)	269	0	600	79.17	74.64
Bicycling for 10 min (days)	255	0	7	0.40	1.21
Time spent bicycling on one of those days (min)	249	0	180	9.40	24.88
Continuous walking for at least 10 min (days)	258	0	7	4.81	2.32
Time spent walking on one of those days (min)	262	0	800	59.76	83.43
*Physical activity—house work, house maintenance and caring for family*					
Vigorous-intensity physical activities around the house (days)	253	0	7	0.56	1.18
Time spent on vigorous-intensity physical activities on one of those days (min)	247	0	600	24.54	65.60
Moderate-intensity physical activities around the house (days)	251	0	7	1.29	1.93
Time spent on moderate-intensity physical activities on one of those days (min)	251	0	420	34.56	60.11
Moderate-intensity physical activities in the house (days)	257	0	7	2.64	2.17
Time spent on moderate-intensity physical activities in the house on one of those days (min)	258	0	420	61.32	62.79
*Physical activity—recreation, sport in spare time*					
Continuous walking for 10 min in spare time (days)	256	0	7	2.61	2.49
Time walking on one of those days (min)	254	0	600	48.19	63.88
Vigorous-intensity sport (days)	252	0	7	1.32	1.74
Time spent on one of those days (min)	250	0	360	39.20	50.85
Moderate-intensity sport (days)	245	0	7	0.98	1.68
Time spent on one of those days (min)	242	0	200	23.07	34.35
*Time spent sitting*					
Time spent sitting on weekday (h)	263	2	40	8.96	6.39
Time spent sitting on off-work day (h)	261	0	42	7.00	6.88

Min—minimal, Max—maximal, M—mean, SD—standard deviation

The second part of the questionnaire consisted of questions related to the methods of traveling to work, for shopping, to places of entertainment and etc. These questions referred to activities which were not carried out in the spare time. Respondends’ answers revealed that they use a means of transport definitely more often (M 5.60; SD ± 1.91) than a bicycle (M 0.40; SD ± 1.21). When it comes to walking it took only 4.81 days (SD ± 2.32) and 59.79 min (SD ± 83.43) in four days (Table 3).

The next questions covered the physical activity in the period of previous 7 days, which was done at home or around home, e.g. housekeeping, taking care for family, doing basic cleaning tasks, gardening. Moderate-intensity physical activity included such works as carrying light loads, washing windows, scrubbing floors, sweeping, raking, cleaning, etc. According to respondends moderate-intensity activities at home were performed most frequently, i.e. 2.64 days (SD ± 2.17) and consumed 61.32 min on average in one of these days. Works around the house of moderate-intensity were performed on 1.29 days (SD ± 1.93) and consumed an average of 34.56 min on one of those days. Vigorous-intensity works around the home (heavy lifting, chopping wood and digging) were undertaken by the respondends definitely less commonly and did not last long. The presented studies indicate that moderate-intensity physical activity lasted longer than vigorous-intensity one and was undertaken approx. twice as common as other types of physical activities (Table 3).

The fourth part of the project concentrated on the recreation, sport, exercise and physical activity in the spare time. This part did not include information about physical activity, which had already been described in the previous sections of the questionnaire. Answers provided by the subjects indicate that walking was the most common form of activity in the spare time—48.19 min on average. Walk was also presented as the most common form of recreation—more than twice a week (M 48.19 min, SD ± 63.88). Vigorous-intensity (aerobic, running, bicycling fast, swimming fast, etc.) and moderate-intensity sports (bicycling at a regular pace, swimming at a regular pace, playing volleyball, etc.) consumed almost the same amount of time—approx. 39.20 min (SD ± 50.85)–23.07 min (SD ± 34.35) per day and were essentially carried out once a week (Table 3).

The last part of the questionnaire contained questions related to the time spent in sedentary behavior. The respondends included time spent at work, at home, while studying and free time. This could be time spent on reading, watching TV, visiting friends etc. This time did not include time spent on sleeping or travelling (using means of transport). The results of the study show that the average amount of time spent in sedentary position during the weekday and time off is circa 7 h (Table 3).

Detailed analysis of the obtained data revealed that the physical activity level in university employees is low (n = 222; 80.43%). Only 14.86% of respondends present moderate physical activity level, whereas 4.71% (n = 13) high level (Table 1). Mean general volume of physical activity was 473.10 MET min/week (SD ± 603.40). The highest mean MET value as associated with physical activity related to transportation 557.72 MET min/week (SD ± 917.14) (Table 4).

**Table 4:** Physical activity expressed in MET-min/week—mean results and standard deviation

Physical activity—mean results (MET)	MIN	MAX	M	SD
Total volume of work-related physical activity	0	5940	350.68	741.23
Total volume of transportation-related physical activity	0	9240	557.72	917.14
Total volume of household-related physical activity	0	6860	376.57	743.63
Total volume of physical activity in spare time	0	3630	473.10	603.40
Total physical activity	0	3836	439.52	480.22

Min—minimal, Max—maximal, M—mean, SD—standard deviation

**Fig. 1:**
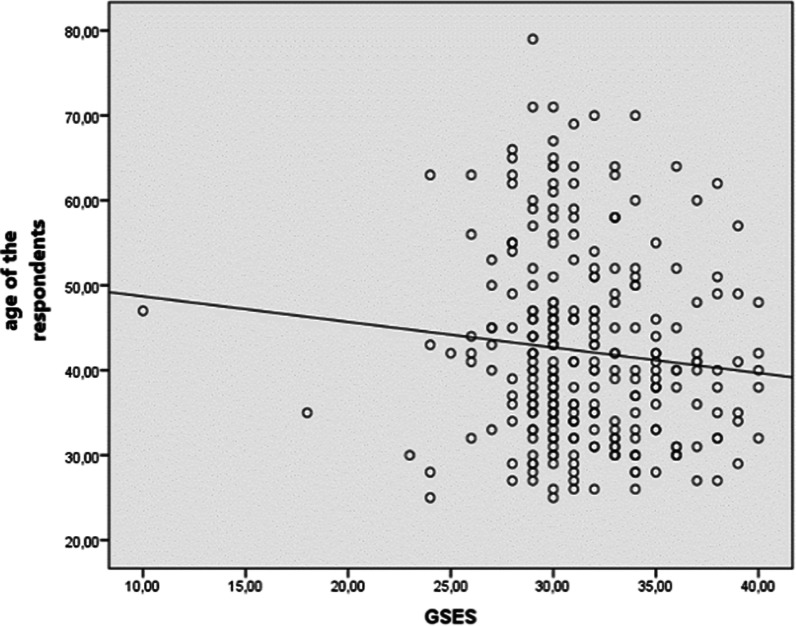
Correlation between age and GSE scale—scatter diagram (rHO = − 0.11; *p* = 0.050)

The analyses performed using Spearman correlation test and independent samples Student t-test did not reveal any relationship between the physical activity and the age (rHO = − 0.01; *p* > 0.5), BMI (rHO = − 0.04, *p* > 0.05). The analysis did not reveal the relationship between undertaking physical activity and academic degree (H_3_ = 3.24; *p* > 0.05). Student t-test was used to verify whether the type of university has any impact on the fact of undertaking physical activity. The results of the study indicate that respondends employed at the Medical University show statistically significant higher level of physical activity than the employees of Technical University (t_267_ = 2.34; *p* < 0.05) (Table 5). The analysis (including OR) showed that an insufficient level of physical activities among the group of the technical university staff has a strong association with the alcohol consumption. The respondends who did consume alcohol experienced a significant decrease in PA (Table 6). Our study did not reveal any relationship between undertaking physical activity and the level of self-efficacy (rHO = 0.06; *p* > 0.05). Further analyses with Spearman correlation test confirmed that the level of self-efficacy decreases with the age of the respondends (rHO = − 0.11; *p* = 0.05) (Fig. 1).

**Table 5:** The results of the Student t-test to determine the differences of the variables—physical activity (in MET-min/week) versus type of the university

Physical activity versus type of university	N	M	SD	t	Df	*p*
Medical University	125	513.37	609.13	234	274	0.020*
Technical University	151	378.38	328.26			

Min—minimal, Max—maximal, M—mean, SD—standard deviation; the Student t-test to determine the significant variables

**Table 6:** The odds ratio value (OR) and its 95% confidence intervals (95% CI) for the factors potentially related to low physical activity

Factor	OR [95% CI]			*p* value
	Medical University	Technical University	All	
Overweight (BMI > 25)^1^	1.42 [0.56–3.59]	0.9 [0.42–2.19]	1.15 [0.63–2.09]	0.657
Gender^2^	1.80 [0.61–5.28]	1.12 [0.48–2.61]	1.32 [0.72–2.42]	0.360
Post	1.35 [0.13–13.87]	1.45 [0.36–5.79]	1.40 [0.43–4.55]	0.574
Alcohol consumption^3^	–	2.01 [0.55–7.31]	3.85 [1.14–13.02]	0.029*
Smoking^4^	0.71 [0.23–2.19]	1.08 [0.33–3.52]	0.87 [0.39–1.95]	0.741
Children^5^	0.87 [0.17–4.45]	4.85 [0.62–37.89]	2.16 [0.63–7.43]	0.223
General assessment of health	4.92[0.61–39.66]	9.96 [1.28–77.47]	7.36 [1.72–31.52]	0.007*

^1^Overweight BMI > 25 versus the other

^2^Males versus females

^3^Alcohol consumption (once a day) versus the others

^4^Smokers versus the others

^5^Number of children > 3 versus the other

^6^General assessment of health (poor) versus the other. differs significantly

^7^General assessment of PA versus alcohol consumption, *p* < 0.05

## Discussion

The progress in economic development leads to a situation where we spend more time working at the expense of physical activity [29]. In May 2013 WHO developed a new policy on prevention and control of non-communicable diseases for 2013–2020. This plan was created to act in four areas, including: addiction to cigarettes and alcohol, unhealthy diet and lack of physical activity [30]. According to many researchers the above-mentioned factors have a considerable impact on morbidity indexes and present a challenge for not only such medical disciplines as public health, but also for other medical and social sciences [31]. The growing problem of maintaining a healthy body mass, lack of physical activity and spreading overweight and obesity epidemic is a great problem of twenty-first century societies. Results of our own studies revealed that albeit over 50% of respondends had normal body mass, the problem of overweight and obesity impacted a significant group of the respondends. These results are consistent with what has been earlier reported by the other researchers [29, 30]. Despite the fact that most authors indicate that obesity is fourfold more common among unqualified workers, the development of civilization goods, industrialization, excessive devotion to transport, availability of fast food, lack of spare time to prepare own meals lead to a situation where the risk of becoming overweight and obese concerns qualified workers, specialist in their own fields [31–33]. Our own data makes it clear that although overweight and obesity were not a dominating feature, yet the analysis of the literature in correlation with our own data shows distinct growing trend [11]. This observation is supported by multiple reports and data including results of WHO statistical analyses [29, 30].

Some research showed that perceived self-efficacy and social support are important determinants of undertaking physical activity [33, 34]. Our results clearly show that academics present very high level of self-efficacy, yet it does not significantly impact their decision to undertake physical activity. Project opposes to some extent the results and observations of other authors [34]. Most research confirms that success in active living depends on the level of self-efficacy [33, 34]. Our results confirmed that the level of self-efficacy among university teachers is high and decreases with age. The lack of correlation between that factor and PA may result from the fact that most of the activities undertaken by the respondends were work-related behaviors. Relatively small percentage of respondends filled their free time with interesting sports activities. Taking into account the results of our project, it might be worth considering the self-efficacy factor in the health projects or training methods dedicated to that group of workers.

The association between the low level of PA and alcohol consumption has been another very interesting result. The opinion regarding that global problem remains unclear. Some study provides evidence of a relationship between moderate alcohol consumption and the increased risk of stroke or artery disease [35]. Other authors suggest that this factor very often accompanies sedimentary lifestyle and in longer perspectives may lead to health problems [36, 37]. For sure that issue requires further research. The statistical analysis of our project did not determine that the age, gender, BMI index have an impact on the low level of physical activity. Our outcomes are in contrast with other projects. Some research suggests that there is a correlation between the level of physical activity and the marital status or gender. The authors of this study concluded that female healthcare workers tend to be more physically inactive because of the childcare and family life [38]. Pettee and co-authors confirmed that married men reported higher median levels of exercise participation in comparison to married women who reported higher level of non-exercise activity [39]. Bergier et al. suggest that there is a co-existence of a high BMI index and a medium level of PA [40]. Our study did not confirm that relationship. But the response rate of the respondends who were classified as a pre-obesity group was also relatively small.

Our study allows classification of the respondends into one of the three physical activity categories: low, moderate and high according to the specified key. The analysis of the collected data revealed that the level of extra-occupational physical activity in academics and research fellows is insufficient and does not exceed 600 MET-min/week. The presented results confirm the ones published by Jasik [41]. Teachers, corporation employees and healthcare professionals present insufficient physical activity level. According to the authors of the above-mentioned paper it can be significantly related to the nature of their work, lack of spare time and lack of adequate motivation to exercise. The result of our studies indicate that despite high self-efficacy level, MET/week ratio is not only insufficient but even threefold lower than in studies carried out by Stanislawska et al. [42], where physical activity index of nursing personnel was 3707, 12 MET-min/week.

The presented results of our own studies confirmed that it is the job-related physical activity, including travelling by means of transport which consumes most of the respondends’ time. Moreover, rapid economical growth and higher salary repeatedly encourage employees to undertake occupational duties at the expense of time which could be spent on the physical activity. Our own study allowed respondends to indicate the most popular form of physical activity. These results clearly show that walking has gained much recognition among researchers and they are consistent with what was reported by the other authors [42]. All activity related to walking are the most readily available form of the physical activity that does not require any additional financial investment. Nevertheless, results of different studies show that it must be carried out with an adequate quantity and frequency to bring any measurable health benefits. Worldwide recommendations, including these issued by WHO, promote 10,000 steps every day. According to many authors such number is unfortunately very hard to achieve by occupationally active subjects [3, 43].

The results of detailed analyses indicated that physical activity level is definitely higher, although not satisfactory, when it comes to the employees of the medical universities in comparison with the employees of the other facilities. Despite small number of publications regarding this issue, results presented in our paper are consistent with what was reported in available literature. Some authors indicate that medical professionals undertook physical activity significantly more often than employees of other professions, including corporations and education field [42–44]. It is obviously associated with a higher health-related awareness and knowledge of factors which disturb this well-being.

Without any doubts, PA improves personal well-being, reduces the risk of many chronic diseases. The lack of physical exercise has clearly been shown as a risk factor of cardiovascular diseases and sudden deaths—this, in turn, affects the economic status of the health care system [45]. However, it should be emphasized that healthcare professionals and teachers of the medical universities should primarily promote the health benefits of the activity behaviors. They should support the efforts of implementing health programs, practices, and policies that facilitate the increase of the level of PA and improve people's health.

## Conclusions

The results obtained for both university groups are not fully satisfactory. The research showed that the university staff, even the medical teachers (professionals in the different fields of medicine) present mainly job-related activity. The self-efficacy test results show that this group of population is able to perform a particular behavior. On the other hand the lack of time for non-professional activities seems to be the biggest problem. What appears positive, however, when it comes to this group of teachers is that the majority of respondends demonstrate a healthy BMI index and evaluate their health condition on a good level. Our statistical analysis did not confirm that factors like gender, age or family status determine the low level of physical activity. Although respondends who drink more than a few times a week evaluate their health condition on the poor level. The association between the alcohol intake, its relation with the insufficient PA would have to be investigated in the future research.

## Study limitations

The study has certain limitations. The first one is a research method. The results obtained from this analysis show that we cannot generalize them in terms of a population because the description is very specific, it is a narrow sample. The other one is the time at which the study has been carried out. According to the recommendations issued by the author of the IPQA scale, the full version of the tool should not be performed during winter. The interview should not be carried out during weeks with drastically bad weather because it includes questions concerning physical work in the garden or riding a bicycle. The analysis of the physical activity level of the respondends presented in our study is therefore seasonal in nature. Another rather important problem, which arose during the project, was the need to make the respondent confirm how many times had he/she performed activities related to physical effort and how long had each of this activity lasted. According to generally accepted rules for IPAQ, the activities taken into account in this questionnaire (related to the transportation or physical work) should last at least 10 min (without any interruptions), as only such continuous physical activity has beneficial effect on the body.

Online surveys offer many advantages over the traditional version of the surveys including access to the specific populations, speed of data access, decreased data collection and costs. It might be also the source of many doubts and problems. The first disadvantage of that method is the sampling issues, and that it might be not a nationally representative group. Unfortunately, the method leads to the situation that it is difficult to estimate how many respondends got the invitation and responded to the questionnaire. Another important concern in data collection is data security. Although Google application is certified by the Federal Security Management Act (FISMA), it is still possible that a hacker can break into the data center.

The last limitation refers to the calculation method of the Body Mass Index (BMI). BMI is quite a common factor used in many different studies in order to allocate the weight of respondends into the few groups: underweight, normal weight, overweight and obese with three categories. The index in the project was calculated on the basis of weight and height self-report. That type of calculations have major limitations including misclassification problem. For sure the BMI does not give any insight into the regional body fat distribution. That self-report method was not an ideal solution, nevertheless, it is still a very simple method used to estimate the health risk of obesity.

## Data Availability

The datasets analysed during the current study, is available on request, from Dr. Katarzyny Kwiecień-Jaguś, katarzyna.kwiecien-jagus@gumed.edu.pl.

## Abbreviations

BMI: Body Mass Index
GSE: Generalized Self-Efficacy Scale
IPAQ: International Physical Activity Questionnaire
MET: Metabolic Equivalent of Task
OR: Odds ratio
PA: Physical activity
WHO: World Health Organization
